# Supervised physical therapy in women treated with radiotherapy for breast cancer**^1^**

**DOI:** 10.1590/1518-8345.0702.2755

**Published:** 2016-08-15

**Authors:** Nara Fernanda Braz da Silva Leal, Harley Francisco de Oliveira, Hélio Humberto Angotti Carrara

**Affiliations:** 2 MSc, Physical Therapist, Centro de Fisioterapia Maria Izabel Garnica Roberto, Pontal, SP, Brazil.; 3 PhD, Professor, Faculdade de Medicina de Ribeirão Preto, Universidade de São Paulo, Ribeirão Preto, SP, Brazil.

**Keywords:** Breast Neoplasms, Radiotherapy, Physical Therapy Specialty

## Abstract

**Objective:**

to evaluate the effect of physical therapy on the range of motion of the shoulders and perimetry of the upper limbs in women treated with radiotherapy for breast cancer.

**Methods:**

a total of 35 participants were randomized into two groups, with 18 in the control group (CG) and 17 in the study group (SG). Both of the groups underwent three evaluations to assess the range of motion of the shoulders and perimetry of the upper limbs, and the study group underwent supervised physical therapy for the upper limbs.

**Results:**

the CG had deficits in external rotation in evaluations 1, 2, and 3, whereas the SG had deficits in flexion, abduction, and external rotation in evaluation 1. The deficit in abduction was recovered in evaluation 2, whereas the deficits in all movements were recovered in evaluation 3. No significant differences in perimetry were observed between the groups.

**Conclusion:**

the applied supervised physical therapy was effective in recovering the deficit in abduction after radiotherapy, and the deficits in flexion and external rotation were recovered within two months after the end of radiotherapy. Registration number of the clinical trial: NCT02198118.

## Introduction

Cancer is a chronic disease characterized by uncontrolled cell growth due to changes in the genetic code. Breast cancer is the second most common type of cancer worldwide and is the most common type of cancer among women. Gynecologic variables, anthropometric variables, history of breastfeeding and alcohol consumption, body composition, and physical activity levels are potential risk factors for this disease^1^ ^-^ ^3^.

Early diagnosis is one of the primary prognostic factors, and the therapeutic choice depends on the clinical stage, anatomopathological characteristics of the disease, and the clinical status of the patient. Local treatment consists of surgery and radiotherapy (RT), whereas systemic treatment consists of chemotherapy (CT), hormone therapy (HT), and biological therapy^3^ ^-^ ^4^.

Postoperative RT may be associated with complications that affect the quality of life of patients^5^. This treatment destroys cancer cells but also affects healthy tissues around the irradiated area and causes vascular lesions. These lesions can evolve to fibrosis and adhesion between the skin and muscles of the chest wall, shoulder, and supraclavicular and axillary cavities^6^. In addition, RT can cause lymphedema, impaired motion of the shoulder, pain, stiffness, and fatigue^7^ ^-^ ^9^.

Few studies have evaluated the effectiveness of physical therapy during RT. A previous study found that physical therapy intervention applied during RT prevented the limitation of the range of motion (ROM) of the shoulder, reduced the incidence of scar tissue adhesion, and improved the quality of life^10^ ^-^ ^11^. Another study reported that physical activity during RT increased shoulder ROM, improved the quality of life and decreased fatigue^5^.

Considering the severity of the possible consequences of RT and the limited number of studies on this topic, the objective of the present analysis was to evaluate the effect of physical therapy intervention during the RT period on the ROM of the shoulder and perimetry of the upper limbs in women treated with radiotherapy for breast cancer.

## Methods

This study was approved by the Research Ethics Committee of the Clinical Hospital of the Ribeirão Preto Medical School, University of São Paulo (Hospital das Clínicas da Faculdade de Medicina de Ribeirão Preto da Universidade de São Paulo-HCFMRP-USP) under Protocol No. 11678/2009.

This clinical, prospective, non-blinded, randomized, controlled study was conducted in the Mastology Clinic and Radiotherapy Service of HCFMRP-USP between November 2009 and March 2012. 

### Sample characteristics

The study population consisted of women treated at the Mastology Clinic and Radiotherapy Service who met the following inclusion criteria: (i) diagnosis of unilateral breast cancer; and (ii) undergoing surgery and RT for breast cancer, conducted according to the therapeutic protocol of the center. The exclusion criteria were patients with orthopedic and/or neurological disorders that limited the movement of the upper limbs, bilateral breast cancer, prior thoracic RT, and the presence of distant metastasis. 

All of the participants were invited to participate in the study by telephone and were successively treated at the Radiotherapy Service with an indication for RT as part of their treatment. Those who met the inclusion criteria and agreed to participate were included in the study and signed an informed consent form. A total of 35 participants were selected. The study group comprised homemakers or women who were out of work because of breast cancer treatment. The participants did not practice physical exercise and were therefore considered sedentary. 

### Evaluation protocol and physical therapy intervention

The participants were evaluated at three different time points: pre-RT (evaluation 1), post-RT (evaluation 2), and two months after the end of RT (evaluation 3). The variables evaluated were the ROM of the shoulder joint and perimetry of the upper limbs. Shoulder ROM was evaluated by assessing flexion, extension, abduction, adduction, and internal and external rotation, which were actively performed by the participants. These measurements were made using a Carci(r) goniometer, and positioning was performed according to the protocol proposed by Marques^12^. Perimetry involved the performance of measurements at six different points: point A - in the metacarpophalangeal joints of the second, third, fourth, and fifth fingers; point B - an imaginary line pointing in the direction of the metacarpophalangeal joint of the first finger; point C - 10 cm below the olecranon; point D - 6 cm below the olecranon; point E - 6 cm above the olecranon; and point F - 10 cm above the olecranon^13^. The subjects remained in a sitting position with the arm resting on the thigh and the forearm supinated. The measurements were bilateral.

The selected participants were randomly divided into two groups: one group was subjected to the evaluations described in the paragraph above and was designated the control group (CG). Another group was subjected to supervised kinesiotherapy of the upper limbs and was designated the study group (SG). The randomization plan was generated using computer software that distributed the participants into the two groups, following the sample size obtained in the sample calculation. The participants were distributed into each group during evaluation 1. The distribution was random but non-blinded. 

**Figure 1:**
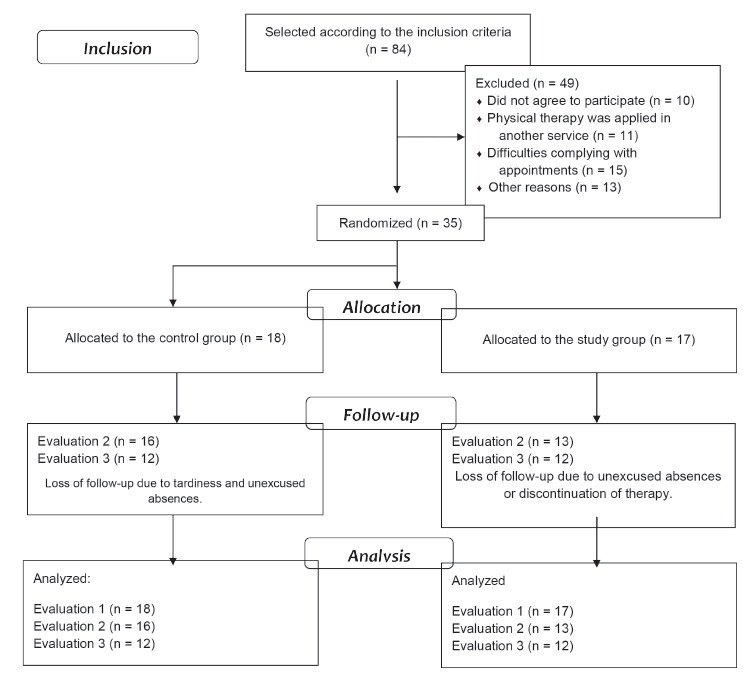
CONSORT diagram: inclusion, allocation, monitoring, and analysis

Figure 1 shows the plans for inclusion, allocation, monitoring, and analysis.

Kinesiotherapy was performed during the RT period on an individual basis and was performed in both limbs and twice a week. The exercise protocol^14^ was performed under the supervision of a physical therapist. Kinesiotherapy consisted of 14 free active exercises for the cervical spine and upper limbs, with a series of 15 repetitions, and was performed in a sitting position. The exercises involved cervical tilt and rotation; elevation, flexion, extension, abduction, adduction, and rotation of the shoulder; and flexion and extension of the elbow and wrist.

### Statistical analysis

Considering an α of 0.05, a test power of 80%, differences in the mean perimetry values of 3.0 cm before and after RT, and a standard deviation of 4.5, a required sample size of 16 was calculated using Power and Sample Size Calculation version software 2.1.31^15^ .

The analysis involved the assessment of the intention to treat (ITT) and included all participants in the study group who were originally allocated by randomization, irrespective of the period of initiation of treatment, discontinuation of therapy, nonadherence to the protocol received, or the use of treatment protocols that differed from the original^16^. 

For the intragroup analysis, an unpaired t-test was used to compare the goniometry results between the ipsilateral and contralateral limbs within the same evaluation. One-way analysis of variance (ANOVA) was used to assess differences in perimetry among the three evaluations. For intergroup analysis, unpaired t-tests were used to evaluate the goniometry results in the ipsilateral limb and differences in perimetry. P-values lower than 5% were considered statistically significant.

## Results

Table 1 shows the characteristics of the participants with respect to age, body weight, type and duration of surgery, and disease staging. 

**Table 1:** The characteristics of the study participants. Ribeirão Preto, São Paulo, Brazil, 2012.

Characteristic		Control group (n = 18)	Study group (n = 17)
Age (years)*		*54.8* ±11.86	55.2 ± 7.14
Body weight (kg)*		70.75 ± 18.24	69.58 ± 8.44
Type of surgery^†^			
	Conservative	13	13
	Radical	4	4
	Axillary lymphadenectomy	11	11
	Sentinel lymph node biopsy	*7*	4
	Plastic surgery	2	-
Duration of surgery (months)*		5.81	4.52
Chemotherapy^†^		15	12
Hormone therapy^†^		13	11
Disease staging^†^			
	0	1	3
	I	3	2
	IIA	4	6
	IIB	4	2
	IIIA	2	3
	IIIB	4	2
	IIIC	-	-
	IV	-	-

*Mean and standard deviation (SD)

† Number of participants

Considering the RT period, an average of 11 supervised physiotherapy sessions should have been conducted in the SG, but only 8 (72.72%) were conducted. 

Radiotherapy was performed according to the protocol in force in the service, in which the irradiated regions were the breast or chest plastron in all 35 participants and the supraclavicular fossa (SCF) in 7 participants from the two study groups. The participants were treated with conformational RT (3DRT) in the residual breast or chest plastron and the SCF, when indicated, for lymphatic drainage regions. Fractionation was 1.8 and 2.0 Gy/fraction (one fraction per day, 5 days per week) using total doses between 45.0 and 50.4 Gy in 25 fractions in the first treatment stage. During tumor bed boost, the prescribed dose was 9-10 Gy in 5 fractions or 1.8-2.0 Gy per fraction (one fraction per day, 5 days per week) and involved only the target volume or quadrant that was previously affected by the tumor. Therefore, the RT duration in both groups was five weeks. The duration of RT was six weeks in cases where tumor bed boost was indicated.

The CG exhibited ROM deficits in external rotation in evaluations 1, 2, and 3, whereas the SG exhibited ROM deficits for flexion, abduction, and external rotation in evaluation 1. The deficit in the abduction was recovered in evaluation 2 in the SG, and the deficits in all movements were recovered in evaluation 3. The intergroup analysis indicated no significant difference in the ipsilateral goniometry results. The goniometry data are shown in Table 2.

**Table 2:** Goniometry of ipsilateral and contralateral limbs: evaluation 1 (T1), evaluation 2 (T2), and evaluation 3 (T3) (mean ± SD). Ribeirão Preto, São Paulo, Brazil, 2012

	Study group (GE)				Control group (CG)				IL* SG vs. CG
	IL*	CL^†^	p		IL*	CL^†^	p		P
Flex^‡^_T1^‡‡^	135.80° ± 17.48	147.40° ± 9.44	<0.05		134.0 ± 26.2	148.3 ± 14.6	<0.05		0.82
Flex^‡^_T2^§§^	140.40° ± 10.41	148.60° ± 7.41	<0.05		139.1 ± 18.1	150.8 ± 14.1	<0.01		0.82
Flex^‡^_T3^||^	143.40° ± 9.76	148.00° ± 5.38	0.22		139.4 ± 15.6	149.2 ± 13.3	<0.05		0.46
									
Ext^§^_T1^‡‡^	39.53° ± 7.18	44.41° ± 8.02	0.07		41.78 ± 9.1	43.3 ± 9.0	0.302		0.42
Ext^§^_T2^§§^	41.46° ± 5.92	43.88° ± 5.52	0.40		37.9 ± 41.8	41.8 ± 8.0	<0.01		0.22
Ext^§^_T3^||^	40.25° ± 5.89	43.17° ± 4.32	0.18		39.3 ± 8.1	40.3 ± 7.0	0.524		0.73
									
Abd^|^_T1^‡‡^	133.20° ± 22.70	147.20° ± 14.08	<0.05		127.9 ± 31.0	146.1 ± 22.8	<0.01		0.57
Abd^|^_T2^§§^	140.80° ± 16.61	149.10° ± 3.13	0.17		132.9 ± 21.8	146.6 ± 20.3	<0.05		0.29
Abd^|^_T3^||^	139.70° ± 14:53	147.30° ± 14.75	0.22		133.0 ± 20.5	149.1 ± 17.9	<0.05		0.37
									
Ad^¶^_T1^‡‡^	28.71° ± 7.88	31.53° ± 8.25	0.32		29.4 ± 13.9	32.8 ± 7.6	0.291		0.85
Ad^¶^_T2^§§^	29.38° ± 7:34	30.31° ± 30.06	0.74		25.81 ± 9.4	31.3 ± 8.6	0.062		0.27
Ad^¶^_T3^||^	28.42° ± 7:33	31.25° ± 9:58	0.42		28.8 ± 8.5	33.3 ± 10.0	0.070		0.92
									
ER^**^_T1^‡‡^	74.88° ± 15.10	83.76° ± 4.70	<0.01		73.0 ± 14.4	81.1 ± 7.4	<0.05		0.66
ER^**^_T2^§§^	75.46° ± 10:45	83.15° ± 6.18	<0.05		72.9 ± 13.0	82.1 ± 9.3	<0.05		0.57
ER^**^_T3^||^	78.33° ± 9.76	82.50° ± 5:38	0.21		70.8 ± 15.4	83.4 ± 9.2	<0.05		0.17
									
IR^††^_T1^‡‡^	74.18° ± 14:33	77.94° ± 09.05	0.37		74.3 ± 12.1	72.9 ± 12.2	0.510		0.97
IR^††^_T2^§§^	73.62° ± 12.02	76.23° ± 7:53	0.51		77.1 ± 9.1	75.1 ± 9.4	0.382		0.39
IR^††^_ T3^||^	76.08° ± 9:46	76.92° ± 7.66	0.81		75.9 ± 7.4	76.3 ± 8.4	0.827		0.96

* IL: ipsilateral limb; † CL: contralateral limb; ‡ Flex: flexion; §Ext: extension; |Abd: abduction; ¶Ad adduction; **ER: external rotation; †† IR: internal rotation; ‡‡ T1: evaluation 1; §§T2: evaluation 2; ||T3: evaluation 3.

The intragroup point-by-point analysis of perimetry indicated no significant differences in perimetry. However, the intergroup analysis indicated a significant difference in point F in evaluation 3. These results are shown in Table 3.

**Table 3:** Difference in perimetry between ipsilateral and contralateral limbs in evaluation 1 (T1), evaluation 2 (T2), and evaluation 3 (T3) (mean ± SD). Ribeirão Preto, São Paulo, Brazil, 2012

		T1	T2	T3		P-value*
Point A	Study group	0.04 ± 0.46	12.12 ± 0.87	12.17 ± 0.81		0.90
	Control group	0.03 ± 0.65	0.20 ± 0.75	0.29 ± 0.62		0.67
	p-value†	0.89	0.78	0.67		
						
Point B	Study group	0.14 ± 0.63	0.15 ± 0.85	0.67 ± 1.15		0.26
	Control group	-0.19 ± 0.84	0.10 ± 0.63	0.29 ± 0.75		0.38
	p-value†	0.32	0.85	0.36		
						
Point C	Study group	0.25 ± 0.98	0.38 ± 1.21	0.75 ± 1.05		0.50
	Control group	-0.17 ± 0.94	-0.03 ± 1.38	0.25 ± 1.27		0.50
	p-value†	0.25	0.40	0.30		
						
Point D	Study group	0.29 ± 0.95	0.42 ± 1.37	0.50 ± 1.22		0.87
	Control group	-0.03 ± 0.88	-0.03 ± 1.04	0.17 ± 1.09		0.89
	p-value†	0.47	0.33	0.47		
						
Point E	Study group	0.43 ± 1.31	0.46 ± 1.68	0.96 ± 1.42		0.61
	Control group	0.36 ± 1.03	0.13 ± 1.23	0.08 ± 1.29		0.98
	p-value†	0.89	0.56	0.13		
						
Point F	Study group	0.32 ± 1.28	0.54 ± 1.64	0.87 ± 0.98		0.58
	Control group	0.17 ± 1.40	0.20 ± 1.31	-0.21 ± 1.17		0.64
	p-value†	0.88	0.55	<0.05		

*Intragroup comparison

†Intergroup comparison

## Discussion

Physical therapy can help reduce pain, fatigue, and lymphedema, and improve muscle strength, shoulder ROM, functional status, and the quality of life of women undergoing treatment for breast cancer^17^ ^-^ ^20^. 

The occurrence of postoperative complications depends on the surgical extension, axillary approach, and application of CT and RT. RT is associated with increased loss of the ROM and muscle strength, lymphedema, fibrosis in the chest wall, and impaired neoformation of lymphatic vessels. Fibrosis and lymphedema are more frequent during SCF irradiation and tumor bed boost. The upper limbs are less impaired when RT excludes the axilla^6^ ^,^ ^9^ ^,^ ^21^ ^-^ ^22^.

The deficit in the ROM observed in the CG and SG in evaluation 1 can be attributed to surgery because ROM restrictions and functional problems in the shoulder may still be present at six months or more after surgery^20^ ^,^ ^23^.

The CG maintained the deficit for external rotation throughout the study period. In the SG, the deficit in the abduction movement was recovered in evaluation 2, and the deficits in flexion and external rotation were recovered in evaluation 3, demonstrating the importance of conducting supervised physical therapy in women undergoing RT for breast cancer.

A study that evaluated the ROM in the shoulder of women before and after RT indicated an increase in the deficit of flexion and abduction in the control group and a decrease in the group subjected to physical therapy^10^. Moreover, the ROM in women who underwent physical therapy improved during the RT period and worsened in women who did not undergo physical therapy^5^. Physical therapy results in a gain in shoulder ROM when applied during RT; this effect can be observed immediately after the end of RT^5^ and persists for up to six months after RT^10^. Our results are consistent with those of previous studies.

Muscles should be at their natural length and have a sufficient ability to glide under adjacent soft tissues (i.e., skin and subcutaneous tissue) to ensure adequate mobility of the joints. Full range of flexion and abduction requires proper functioning of the major and minor pectoral, latissimus dorsi, teres major, subscapularis, and rhomboid muscles. Adequate functioning of the serratus anterior muscle is also required for the upward rotation of the scapula. For external rotation, the pectoralis major, latissimus dorsi, teres major, and subscapularis muscles must be at their natural length and be able to glide^21^.

Because of their origin and insertion, the pectoral and serratus anterior muscles are approached and can be damaged during surgery for breast cancer. Furthermore, these muscles are located in the areas indicated for RT^6^. Therefore, the movements used to recruit these muscles may be adversely affected by the adhesion and fibrosis caused by RT^5^ ^,^ ^10^ ^,^ ^19^ ^,^ ^24^. 

The analysis of the mean difference in the upper limb perimetry at each study point indicated no significant differences in the intragroup comparison. Although intergroup analysis indicated a significant difference in point F in evaluation 3, this result indicates intergroup differences but not the presence of lymphedema, as shown in Table 3. The analysis of mean perimetry values indicated that the participants did not present with lymphedema after surgery and did not develop this complication during RT or two months after its completion. The same result was found in another study, wherein lymphedema was not observed even six months after the completion of RT^10^.

The risk of the onset of lymphedema is associated with several factors, including radical surgery, extent of axillary dissection, and application of RT. However, pre-existing lymphatic insufficiency of genetic and traumatic origin may also be responsible for the emergence of lymphedema. After axillary lymphadenectomy , the body adjusts to compensate for the removal of lymph nodes to allow for the transport of lymph, thereby preventing the development of lymphedema^9^ ^,^ ^24^ ^-^ ^25^.

The exercises used in this study were free active exercises consisting of a series of 15 repetitions and were performed twice a week during the RT period. These exercises were intended to maintain the movement of joints and soft tissues, minimize the loss of flexibility and the formation of contractures and ensure early rehabilitation^19^. The supervised kinesiotherapy helped recover the deficit in the shoulder ROM between the ipsilateral and contralateral limbs.

The limitations of the present study include the non-blinded nature of the study, the performance of all stages of research (i.e., patient selection and enrollment, randomization of the study groups, evaluation and implementation of interventions) by the same researcher, difficulty in recruitment and protocol adherence of the sample study, and the fragile emotional state of the participants, leading to the discontinuation of therapy before the completion of the study.

Notwithstanding these limitations, the results of this study contribute to the practice of evidence-based physical therapy. Supervised physical therapy is beneficial to patients treated with RT for breast cancer, which was demonstrated by recovery of the shoulder ROM. Therefore, supervised physical therapy should be encouraged and applied in the RT period to prevent and treat possible complications of the upper limbs. 

## Conclusion

Supervised physical therapy that targets the ROM of shoulders of women treated with RT for unilateral breast cancer helps increase the ROM of flexion, abduction, and external rotation. The deficit in the abduction was recovered after RT, and the deficits in flexion and external rotation were recovered two months after the end of RT. The physical therapy protocol applied did not change the upper limb perimetry, a result that is consistent with the fact that the participants did not have lymphedema and did not develop this condition during the study period. These results indicate the need to perform this type of physical therapy in patients treated with RT for breast cancer.
